# Genetic, Genomics, and Responses to Stresses in Cyanobacteria: Biotechnological Implications

**DOI:** 10.3390/genes12040500

**Published:** 2021-03-29

**Authors:** Corinne Cassier-Chauvat, Victoire Blanc-Garin, Franck Chauvat

**Affiliations:** Institute for Integrative Biology of the Cell (I2BC), Université Paris-Saclay, CEA, CNRS, 91198 Gif-sur-Yvette, France; corinne.cassier-chauvat@cea.fr (C.C.-C.); victoire.blanc-garin@cea.fr (V.B.-G.)

**Keywords:** *Synechocystis* PCC 6803, *Synechococcus* PCC 7942, *Synechococcus* PCC 7002, biodiversity, genotype-phenotype relationships, conjugation, transformation, RSF1010 plasmid, neutral cloning sites

## Abstract

Cyanobacteria are widely-diverse, environmentally crucial photosynthetic prokaryotes of great interests for basic and applied science. Work to date has focused mostly on the three non-nitrogen fixing unicellular species *Synechocystis* PCC 6803, *Synechococcus* PCC 7942, and *Synechococcus* PCC 7002, which have been selected for their genetic and physiological interests summarized in this review. Extensive “omics” data sets have been generated, and genome-scale models (GSM) have been developed for the rational engineering of these cyanobacteria for biotechnological purposes. We presently discuss what should be done to improve our understanding of the genotype-phenotype relationships of these models and generate robust and predictive models of their metabolism. Furthermore, we also emphasize that because *Synechocystis* PCC 6803, *Synechococcus* PCC 7942, and *Synechococcus* PCC 7002 represent only a limited part of the wide biodiversity of cyanobacteria, other species distantly related to these three models, should be studied. Finally, we highlight the need to strengthen the communication between academic researchers, who know well cyanobacteria and can engineer them for biotechnological purposes, but have a limited access to large photobioreactors, and industrial partners who attempt to use natural or engineered cyanobacteria to produce interesting chemicals at reasonable costs, but may lack knowledge on cyanobacterial physiology and metabolism.

## 1. Introduction

Cyanobacteria are ancient Gram-negative prokaryotes that perform oxygenic photosynthesis and are phylogenetically close to recently discovered non-photosynthetic bacteria termed Melainabacteria and Sericytochromatia [1,2]. Cyanobacteria are regarded as the producer of the atmospheric oxygen (O_2_) of Earth [3] and the ancestors of the plant chloroplast [4]. Cyanobacteria capture solar energy at high efficiencies (3–9%) [5] to power up their efficient photoautotrophic metabolism, which fixes huge amounts of inorganic carbon (CO_2_, NaHCO_3_, and Na_2_CO_3_) and nitrogen (N_2_, NH_4_, NO_2_, NO_3_, or urea) [6,7], into an enormous biomass [8] that supports a large part of the food chain. 

**Figure 1:**
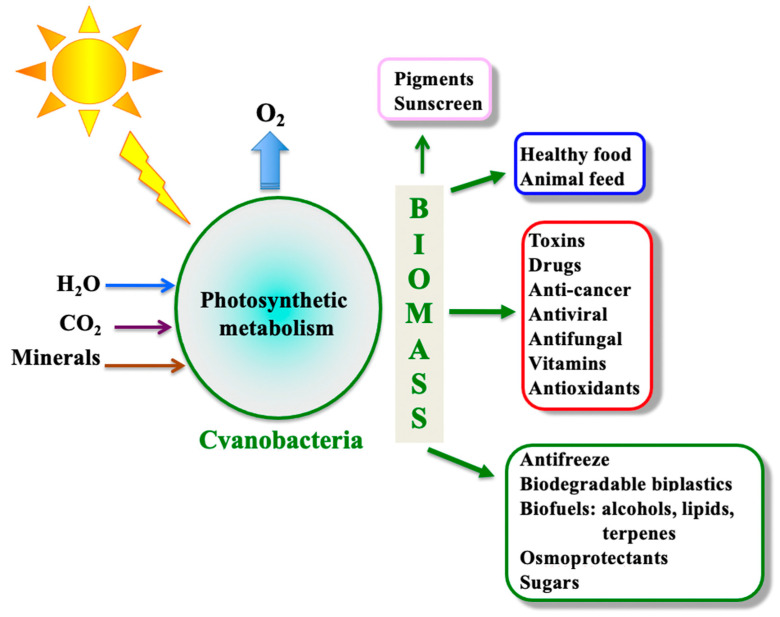
Cyanobacteria can synthesize a wealth of biotechnologically interesting products.

By colonizing aquatic ecosystems (fresh, brackish, and marine waters) and soils (including deserts) of our planet, cyanobacteria are inevitably exposed to multiple stresses such as solar ultraviolet radiations and variations in light intensity and quality, inorganic-nutrients availabilities, temperatures (high and low), salinity, pH (acidic and basic), drought, and pollutants (herbicides and heavy-metals). In addition, cyanobacteria are involved in numerous interactions with competitors, predators, or symbiotic hosts [9]. Consequently, it is not surprising that cyanobacteria have evolved as a widely diverse organisms, which are of high interest for basic and applied research [10]. They display various metabolisms and morphologies [11,12], and numerous species can differentiate cells, akinetes (spores) and/or heterocysts, which are dedicated to growth or survival under adverse conditions [13,14]. Thus, cyanobacteria are good model organisms to study the impact of environmental conditions on the physiology, metabolism, and morphology of microbial cells. Furthermore, cyanobacteria synthesize a wide variety of bioactive metabolites (Figure 1), many of which being of interest for human health [15,16,17,18], and they are regarded as promising cell factories for the production of chemicals (fuels and biodegradable bioplastics) from highly abundant natural resources: solar energy, water (not necessarily potable), CO_2_, and minerals, thanks to their active photosynthesis and the synthetic biology tools of model species [10,19,20]. 

Most of our knowledge on cyanobacteria came from studying the three non-nitrogen fixing, unicellular cyanobacteria *Synechocystis* PCC 6803, *Synechococcus* PCC 7942 (formerly named “*Anacystis nidulans*”) and *Synechococcus* PCC 7002 (formerly named “*Agmenellum quadruplicatum* PR6”) that are (i) straightforward to culture under laboratory conditions, (ii) amenable to genetic manipulation, and (iii) freezable for long-term storage. In this review, we will summarize the genetic and physiological properties of these models, emphasizing on their tolerance to stresses, and the recent progress in their genetics. We will also put forward that in spite of more than three decades of intensive research the genomes of these models still contain a large number of genes and small RNAs (sRNAs) of unknown function. Furthermore, many of the genes annotated “by sequence analogy” with those genes characterized in intensively studied, non-photosynthetic, models such as *Escherichia coli* might in fact have a different function in cyanobacteria. This situation makes comparative genomics and metabolic modeling difficult. Consequently, we will discuss that to better understand and exploit the wide biodiversity of cyanobacteria, strong efforts should be put in large-scale analysis of genes and sRNAs functions in model cyanobacteria. Finally, we will emphasize that we need to identify and thoroughly study new cyanobacteria endowed with natural properties of interest for basic or applied researches, and test whether the synthetic biology tools developed for model strains can be used to facilitate the engineering of these newly identified cyanobacteria so as to turn their promises into industrial realities. 

## 2. Cyanobacteria Being Inevitably Exposed to Photo-Oxidative Stress Have Developed the Evolutionary-Conserved Glutathione System

Having evolved the oxygenic photosynthesis [21], cyanobacteria were the first organisms to be exposed to the toxic reactive oxygen species (ROS): singlet oxygen (^1^ O_2_), superoxide anion (O_2_^−^), hydrogen peroxide (H_2_O_2_), and hydroxyl radical (OH) [22] that are generated when the light-driven electron transport exceeds what is needed for nutrients assimilation [23]. Accordingly, cyanobacteria represent a major source of ROS in natural aquatic environments [24]. 

To cope with ROS, cyanobacteria have developed the glutathione system [3]. It comprises the glutathione tripeptide (gamma-glutamyl-cysteinyl-glycine) and numerous glutathione-dependent enzymes [25], which have been conserved during evolution [26,27,28,29]. Glutathione is abundant in all organisms (concentration ranging from 0.1 to about 10 mM) where it plays pleiotropic roles in cell life and resistance to stresses [25,30]. Supporting the notion that cyanobacteria have evolved the glutathione system, the glutathione synthesis enzyme GshB and several glutathione transferases of the model cyanobacterium *Synechocystis* PCC 6803 were shown to play a prominent role in the tolerance to O_2_ and photo-oxidative stress [31,32]. 

Glutathione occurs under two redox forms: the reduced (GSH) and the oxidized, dimeric (GSSG) forms. GSH, the major form, maintains the intracellular compartment in a reduced state. It provides electrons to the GSH-dependent enzymes glutaredoxins and glutathione transferases that operate in defenses against photo-oxidative stress and heavy metal pollutants [32,33,34]. These toxic-only metals inactivate many enzymes, by reacting with the thiol-group of their cysteines and/or displacing their normal metal cofactors [35]. To protect cells from metals, GSH can chelate them outside the cells under the form of large complexes that cannot penetrate into the cells [36]. GSH can also form a Fe(II)GSH complex, which likely supplies Fe for the synthesis of the Fe cofactor or (Fe-S) clusters of many metalloenzymes operating in photosynthesis, respiration, or other cellular functions [30,37,38].

Oxidative stress can also generate disulfides bridges between two cysteinyl-residues of either the same or two proteins, or a protein and a molecule of glutathione, which otherwise affect protein activity [33,39]. The glutathionylation/deglutathionylation process has been studied in the best studied unicellular cyanobacterium *Synechocystis* PCC 6803 where about 400 proteins can be glutathionylated in vitro [40]. These proteins participate in a wide range of cellular and metabolic processes including carbon and nitrogen metabolisms, cell division, stress responses and hydrogen production [40]. The glutathionylation/deglutathionylation control of the antioxidant peroxiredoxin II, the mercuric reductase, the AbrB2 transcription factor, and the metabolic enzyme 3-phosphoglycerate dehydrogenase was confirmed by biochemical studies with the purified recombinant proteins [34,40,41]. Attesting its importance for living organisms, the redox glutathionylation/deglutathionylation process and the glutathione transferase and glutaredoxin enzymes involved in its control have been conserved during evolution from cyanobacteria to plants and human [26,29,40,42,43].

Upon cell detoxication, GSH is oxidized for the glutathione disulfide form, GSSG, which can be reduced back to GSH by various factors, such as the NADPH-using glutathione reductase enzyme (GR), which occurs in most cyanobacteria [25] and other aerobic organisms [27,44], except the model cyanobacteria *Synechocystis* PCC 6803 [45] and *Synechococcus* PCC 7002 [25].

## 3. Cyanobacteria Possess Widely Diverse Genomes That Contain a Wealth of Unknown Genes and Poorly Characterized Multigene Families

The first articles reporting the sequence of an entire bacterial genome (*Hemophilus influenzae*, 1,830,137 bp; *Mycoplasma genitalium*, 580,070 bp) appeared in 1995 [46,47]. Shortly thereafter, the sequence of the 3,573,470-bp chromosome of the best studied unicellular cyanobacterium *Synechocystis* PCC 6803 was reported [48]. Currently, about 2000 complete or draft sequences of cyanobacterial genomes are accessible in public data bases such as the DOE joint genome institute (https://genome.jgi.doe.gov/portal/, accessed on 15 March 2021) and the Microbial Genome Database for Comparative Analysis (http://mbgd.genome.ad.jp/, accessed on 15 March 2021). This number is steadily increasing in the frame of metagenomic analyses [49,50,51,52]. 

The genome of cyanobacteria differs in size, ranging from 1.44 Mbp [53] to 12.07 Mbp [54], and GC content (30–60%), probably as a result from gene gains and losses [55] transferred by plasmids, insertion sequences [56], and/or cyanophages [57,58,59]. Cyanophages infecting marine cyanobacteria contain genes important for photosynthesis, which could facilitate their transfer among marine cyanobacteria [60]. Instances of horizontal gene transfer are inferred on the basis of observations of patchy phylogenetic distribution and/or atypical nucleotide composition of several genes [55,59].

All cyanobacteria possess a circular chromosome and, depending on the species, a linear chromosome (rarely) and/or one to several circular plasmids (frequently), excepted the marine species UCYN-A, *Prochlorococcus* and *Synechococcus*, which have a small chromosome (1.44–2.7 Mbp), but no plasmids [53,61]. Some cyanobacterial plasmids contain genes likely involved in important processes, such as cysteine biosynthesis [62], thermal tolerance [63], resistance to metal stress [64], or DNA repair [58]. The marine cyanobacterium *Acaryochloris marina* MBIC1101, which has a 6.5 Mbp circular chromosome, has 25% of its genes located on its nine large (≥100 kbp) single-copy plasmids [65].

An unusual genome organization was observed in two strains of the unicellular genus *Cyanothece*. The genome of *Cyanothece* ATCC51142 (5.46 Mbp) harbors two chromosomes (one circular of 4.39 Mbp; and one linear of 0.4 Mbp) and four plasmids (10–39 kbp) [66,67]. *Cyanothece* PCC 7822 has a genome of 7.84 Mbp that comprises one circular chromosome (6.09 Mbp), three linear chromosomal elements (880, 474, and 14 kbp) and three plasmids [67]. These findings are interesting because a few bacteria possess a linear chromosome, such as the (non-photosynthetic) soil bacteria of the genus *Streptomyces*, which synthesize a wide variety of bioactive secondary metabolites [68], like cyanobacteria [16,17,69]. In *Streptomyces*, the linear chromosome exhibits a remarkable genetic organization with grossly a central conserved region flanked by variable chromosomal arms [70]. Based on these observations, it would be interesting in the future to compare the plasticity of linear versus circular chromosomes of cyanobacteria. 

## 4. Interest and Current Limitations of Comparative Genomics

The comparative analysis of cyanobacterial genomes allows to determine which genes are present in any particular genome and which ones are absent. These findings serve to determine the pan-genome, which describes the entire genes set of all cyanobacteria analyzed. It includes (i) a core-genome, which comprises the sets of genes that exist in each of the strains analyzed [71] and (ii) the dispensable-genome, which comprises the genes present in a subset of the studied strains and species-specific genes [72]. For such analyses, strains can be selected on the basis of their natural habitats [73], their physiology and/or morphology [74], and/or their phylogenetic position determined by 16S-rRNA sequence comparison [75]. Such comparative genome analyses revealed habitat-specific (or heterocyst-specific) cyanobacterial proteins [72]. It was also revealed that cyanobacteria occupy a unique position among prokaryotes as a hub between anaerobes and obligate aerobes [76] and that the earliest cyanobacteria were small and unicellular, while filamentous forms appeared shortly afterwards [77]. Comparative genome studies were also used to discuss plastid evolution [78]. When applied to two closely related cyanobacteria, comparative genomics can be used to predict the behavior of one strain relative to the other one. As a rare example of such comparison, *Synechocystis* PCC 6803 was found to be more resistant to zinc excess than *Synechocystis* PCC 6714 as it was predicted [79]. Comparative genomic information is also crucial for genome-based reconstruction of an organism’s metabolism as done for *Synechocystis* PCC 6803 to predict which metabolic reaction or pathway should be engineered to increase the production of biotechnologically interesting chemicals [80,81,82,83,84,85]. 

### 4.1. Danger of Genome Annotation Based Only on Sequence Comparison

Generating robust and accurate genome-scale models (GSMs) is an iterative process dependent on the expansion and updating of draft simulation models with available experimental data obtained after measurement of fluxes and metabolic pool sizes [80,86,87]. However, GSMs are largely based on gene function predicted “by sequence analogy” with genes characterized in other model organisms, which may behave differently in the studied organism [88]. In *Synechocystis* PCC 6803, the best studied model, only ca. 1050 coding sequences (~30%) have assigned functions [80,89], which may turn out to differ from those determined in well-studied heterotrophic models. For examples, the *Synechocystis* PCC 6803 LexA protein regulates the expression of genes involved in carbon assimilation [90], not DNA repair as it occurs in *E. coli*. Furthermore, the NAD(P)H dehydrogenase transcription factor (NdhR, Sll1594), homologous to the proteobacterial regulators of the Calvin−Benson−Bassham metabolic pathway (CbbR, hereafter referred to as Calvin cycle), does not regulate the transcription of Calvin cycle genes [91]. Instead, NdhR regulates numerous genes involved in the transport and assimilation of inorganic carbon (CO_2_ and bicarbonate) [6,92,93].

In addition, a single cyanobacterial protein can have more than one function. The fructose-1,6/sedoheptulose-1,7-bisphosphatase (FBP/SBPase) operates in both the Calvin cycle and gluconeogenesis [94]. The CrtLdiox protein is also dual-function enzyme with both lycopene cyclase and dioxygenase activity [95]. The IsiA protein is active in both the light-harvesting ability of PSI and the photoprotection of PSII [96]. The AgrE enzyme catalyzes two sequential reactions in arginine catabolism, in converting arginine to ornithine, and then ornithine into proline [97,98]. The ApalaDH enzyme transforms pyruvate to L-alanine, L-alanine back to pyruvate, and glyoxylate to glycine [99]. The sucrose-synthesis SPS enzyme has sucrose-phosphate synthase and sucrose-phosphate phosphatase activities [100]. The KatG enzymes have both catalase and peroxidase activities, which are also capable of oxidizing chloride, bromide, and iodide compounds [101,102]. 

### 4.2. Identification of Essential Genes at the Level of a Whole Genome for a Better Understanding of the Genotype-Phenotype Relationships

To use genomic data for reconstruction of an organism’s metabolism it is also important to identify the comprehensive set of genes that are essential to cell growth in well-defined conditions. This has been addressed using transposon mutagenesis in *Synechocystis* PCC 6803 [103,104] and *Synechococcus* PCC 7942 [105,106,107,108]. For the same purpose, the construction of a whole genome library of gene insertion plasmids has been undertaken in *Synechocystis* PCC 6803 [7]. These works will certainly contribute to decrease the high number of proteins with still unknown function. In *Synechocystis* PCC 6803, transposon mutagenesis has been useful to study (i) photosynthesis [109,110,111], (ii) the transport of CO_2_ or bicarbonate [112,113], (iii) the production of the poly-3-hydroxybutyrate (PHB) biodegradable bioplastic [114], and (iv) the NADPH:plastoquinone oxidoreductase complex operating in plastoquinone reduction and cyclic electron transfer (CET) around photosystem I [115,116,117,118,119,120,121]. Transposon mutagenesis has been also useful in *Synechococcus* PCC 7942 to identify a gene in fatty acid production [122]. 

### 4.3. Importance of Deciphering the Selectivity/Redundancy of Multiple Gene Families

To generate robust and predictive model of the metabolism of cyanobacteria, it is also important to unravel the redundancy/selectivity of multigene families, such as those that code for the stress-responsive redox proteins ferredoxins, glutaredoxins, and glutathione transferase. 

Ferredoxins (Fed), are small (acidic) proteins present in most organisms. They use their iron-sulfur cluster (Fe-S) to distribute electrons to various metabolic pathways involved in nutrients assimilation [123]. The best studied cyanobacterium *Synechocystis* PCC 6803 possess nine Feds. The Fed1-6 proteins possess a (2Fe-2S) center, while Fed7 harbors a (4Fe-4S) cluster. In contrast both Fed8 and Fed9 have two clusters: (3Fe-4S) (4Fe-4S) and (4Fe-4S) (4Fe-4S), respectively. The highly abundant Fed1 protein, essential to photosynthesis [124,125], is encoded by a light-inducible gene [126]. The low-abundant Fed2-Fed9 proteins are encoded by stress-responsive genes (light, carbon, herbicides, or heavy metals), which have differential importance (crucial/dispensable) for the photoautotrophic growth and/or the resistance to stresses [45,123,125,127]. Fed1, Fed7, and Fed9 participate in a ferredoxin-glutaredoxin-thioredoxin crosstalk pathway that operates in the protection against oxidative and metal stresses [45]. Fed7, but not Fed9, interacts with a DnaJ-like protein, an interaction that has been strengthened in photosynthetic eukaryotes in the form of a Fed7-DnaJ fusion protein [123]. Fed7 also has a regulatory role under photooxidative stress [128]. Conversely, Fed9, but not Fed7, interacts with the Flv3 flavodiiron protein involved in the photoreduction of O_2_ to H_2_O [123]. Other ferredoxins partners [129,130] should be studied to better understand the selectivity/redundancy of ferredoxins in *Synechocystis* PCC 6803. Attesting their importance for the life of cyanobacteria, the *fed* genes were highly conserved in cyanobacteria. For example, the other models *Synechococcus* PCC 7002 and *Synechococcus* PCC 7942 have, respectively, nine and six *fed* genes, while the symbiotic strain *Acaryochloris marina* MBIC11017 endowed with a large genome (8.36 Mb) possesses sixteen *fed* genes [123]. 

Like ferredoxins, the evolutionary-conserved enzymes glutaredoxins (Grxs) are widely distributed in cyanobacteria [25,131]. Grxs use electrons provided by glutathione (GSH), or the thioredoxin reductase enzyme [45,132], to reduce the oxidative-stress-generated disulfides occurring between two cysteinyl-residues of either the same or two proteins, or a protein and a molecule of glutathione, which otherwise affect protein activity [33,39]. The dithiol Grxs, which possess a CXXC redox center (C and X stand for cysteine and any other amino acid, respectively), catalyze the reduction in protein disulfides or glutathione–protein mixed disulfides (the latter activity is named “deglutathionylation”). The monothiol Grxs, which have a CXXS redox center (S stands for serine), operates in iron sensing and trafficking the biogenesis of iron–sulfur clusters of proteins and deglutathionylation [30]. All cyanobacteria possess a monothiol Grx-encoding gene and a variable number of dithiol Grx genes [25]. *Synechocystis* PCC 6803 possesses three Grxs, which are all dispensable to the standard photoautotrophic growth [25,133]. The dithiol enzymes Grx1 and Grx2, which can interact together [45], operate in tolerance to H_2_O_2_ [45,133], arsenate [134,135], selenate [45], mercury, and uranium [34]. Grx1 can reactivate the oxidized (glutathionylated) form of the mercuric reductase enzyme by catalyzing its deglutathionylation [34]. The monothiol Grx3 (CGFS redox center) enzyme forms a homodimer bridged by a glutathione-ligated (2Fe-2S) cluster [26]. This feature, which has been conserved in Grx3 orthologs from cyanobacteria to plants and mammals, likely operates in Fe sensing and distribution of (2Fe-2S) cluster [26,30,136].

Another important multiple genes family encodes the evolutionary-conserved glutathione transferase (GT) enzymes, which can conjugate glutathione (GSH) on diverse toxics (oxidants, chemicals, and heavy metals) thereby generating water-soluble complexes that can then be degraded or excreted out of the cell [33,137,138]. GST also operate in the glutathionylation/deglutathionylation process [42,43]. Glutathione-S-transferases (GST) are commonly divided in three different families: (i) cytosolic GSTs (the largest family), (ii) mitochondrial GSTs, and (iii) microsomal (membranous) GSTs designated as MAPEGs (membrane-associated protein involved in ecosanoïd and glutathione metabolism) [139]. Little is known about GSTs in cyanobacteria though they are regarded as having evolved the oxygen-generating photosynthesis, as well as GSH and GSH-dependent enzymes to protect themselves against the toxic ROS (reactive oxygen species) massively produced by their active photosynthesis [3]. Based on phylogenetic tree analyses, 12 GST classes were identified in cyanobacteria [140,141]. Cyanobacterial GSTs have been studied mostly in *Synechocystis* PCC 6803, which possesses six GST, namely, Sll0067, Sll1147, Sll1545, Sll1902, Slr0236, and Slr0605 [142,143,144]. While the role of Sll1902 and Slr0605 is still unknown, Sll1545 and Slr0236 were shown to operate in the protection against photo-oxidative stress triggered by high light or H_2_O_2_ [32]. Sll1147 plays a prominent role in tolerance to membrane stresses triggered by heat, cold, and lipid peroxidation [29]. Sll0067 operates in the protection against methylglyoxal (MG), a toxic metabolite by-product of the catabolism of sugars, lipids, and amino-acids, which causes diabetes in human. Sll0067 catalyzes the conjugation of GSH with MG, the first step in MG detoxification catalyzed by the glyoxalase enzymes [145]. 

## 5. A Few Cyanobacteria Are Currently Amenable to Gene Manipulation, Leaving the Wide Biodiversity of Cyanobacteria Largely Unexplored

Historically, three non-nitrogen fixing unicellular cyanobacteria *Synechocystis* PCC 6803 (euryhaline strain), *Synechococcus* PCC 7942 (freshwater strain formerly named “*Anacystis nidulans*”) and *Synechococcus* PCC 7002 (marine strain formerly named “*Agmenellum quadruplicatum* PR6”) were chosen as model because they possess a simple morphology [146,147], a small genome (see above) and the important natural capability to be genetically transformed (see below). In addition, the freshwater strain *Anabaena* (*Nostoc*) PCC 7120 was also chosen as a genetically manipulable representative of filamentous (pluricellular) cyanobacteria [148]. This latter model is well used to analyze the differentiation of heterocysts, the cells dedicated to the fixation of atmospheric nitrogen, and their communication with vegetative cells [14], which are not the focus of the present review. 

Although these four species are phylogenetically distant, they represent only a limited part of the wide biodiversity of cyanobacteria. This is evident from the comparison of the size and organization of their genomes (2.7–7.2 Mbp, absence of linear chromosome) with those of many other cyanobacteria (1.44–12M bp see above, presence of a linear chromosome in a few species). 

New rapidly growing unicellular cyanobacteria have been recently described, namely, the freshwater strains *Synechococcus* UTEX 2973 [149], *Synechococcus* PCC 11801 [150], and *Synechococcus* PCC 11802 [151] and the marine strain *Synechococcus* PCC 11901 [80,152]. *Synechococcus* PCC 11901 tolerates high temperatures, up to 43 °C, which are non-optimal for *Synechococcus* UTEX 2973 [150,153], *Synechococcus* PCC 11801 [150,151], and *Synechococcus* PCC 7942 [154], and even detrimental to *Synechocystis* PCC 6803 [29,155]. *Synechococcus* PCC 11801 [150], *Synechococcus* PCC 11802 [151], and *Synechococcus* PCC 11901 [152] are naturally transformable, unlike *Synechococcus* UTEX2973 [150]. The latter strain was made transformable by cloning the *Synechococcus* PCC 7942 *pilN* gene encoding the Tfp pilus assembly protein [156]. 

Unfortunately, *Synechococcus* UTEX2973, *Synechococcus* PCC 11801, and *Synechococcus* PCC 11802 are very closely related to the freshwater model *Synechococcus* PCC 7942, in sharing, respectively, 99.8% and about 83% of genome identity with it [149,150,151,157]. Similarly, *Synechococcus* PCC 11901 shares 96.7% sequence identity with the marine model *Synechococcus* PCC 7002 [87,152]. Thus, future studies carried out with these new model strains will not significantly increase our understanding of the cyanobacterial biodiversity. 

## 6. Properties of the Intensively Studied Unicellular Cyanobacteria *Synechocystis* PCC 6803, *Synechococcus* PCC 7942, and *Synechococcus* PCC 7002

The three non-nitrogen fixing models *Synechocystis* PCC 6803, *Synechococcus* PCC 7942, and *Synechococcus* PCC 7002 have specific physiological properties that can influence their potential to serve as cell factories for biotechnological projects. *Synechocystis* PCC 6803 in being one of the few cyanobacteria capable to grow under photoautotrophic, mixotrophic, or heterotrophic conditions [147] has become the best studied cyanobacterium. It serves as model to study the photoautotrophic metabolism [6,7,93], the crucial importance of the carbon/nitrogen metabolic balance [6,93], and the responses to stresses [35]. Such investigations are also carried out, to a lesser extent, with *Synechococcus* PCC 7002 and *Synechococcus* PCC 7942. The latter model is well used to investigate cell division [158], the carbon/nitrogen metabolic crosstalk [93], the circadian rhythm [159], and the biogenesis of the CO_2_-concentrating carboxysome organelle [160]. 

### 6.1. Genome Organization in the Intensively Studied Unicellular Cyanobacteria Synechocystis PCC 6803, Synechococcus PCC 7942, and Synechococcus PCC 7002

*Synechocystis* PCC 6803, the best studied model, harbors a 3.57 Mbp circular chromosome [48], which has a copy number of about 10–22 per cell [161,162], and seven plasmids of sizes 2.3 [163,164], 2.4 [165], 5.2 [166], 44, 103, 106, and 120 kbp [167]. The ploidy of the three smaller plasmids 2.3–5.2 kbp was shown to be similar and increased from 3 to 8 copies per chromosome in cells reaching the stationary phase of growth. In contrast, the ploidy of the four larger plasmids, ranging from ∼0.3 to 1.2 per chromosome depending on the studied plasmid, varied little with the growth phase [168]. The 5.2 kbp plasmid appeared to be dispensable to the standard photoautotrophic growth of *Synechocystis* PCC 6803 [169]. In contrast, large plasmids appeared to be essential (cells could not lose these plasmids), likely because they encode several toxin–antitoxin systems mediating plasmid maintenance [170,171] and they operate in beneficial functions such as potassium transport [172], copper resistance [173], and the metabolic tricarboxylic acid cycle [174]. 

The other model cyanobacteria *Synechococcus* PCC 7942 and *Synechococcus* PCC 7002 possess a circular chromosome of, respectively, 2.69 Mbp and 3.0 Mbp, each occurring at two to five copies per cell [175,176,177]. In addition, *Synechococcus* PCC 7942 has two plasmids of 7.84 [178] and 46.4 kbp [179], while *Synechococcus* PCC 7002 contains six plasmids [180] from 4.8 [181] to 186 kbp. The 7.84 kbp plasmid of *Synechococcus* PCC 7942 is not essential to its photoautotrophic growth [182,183]. In comparison, the well-studied filamentous cyanobacterium *Anabaena* (*Nostoc*) PCC 7120 has a 7.2 Mbp genome that comprises a circular chromosome of 6.41 Mbp and six plasmids of sizes 408.10, 186.614, 101.96, 55.41, 40.34, and 5.58 kbp [184].

### 6.2. Physiological Properties of Synechocystis PCC 6803, Synechococcus PCC 7942, and Synechococcus PCC 7002 and Biotechnological Implication

In agreement with them having different genome size and organization, the three model cyanobacteria also show different physiologies. *Synechococcus* PCC 7002, which has a doubling time of ~2.6 h [185], grows 2–7 times faster than *Synechococcus* PCC 7942 and *Synechocystis* PCC 6803 depending on the environmental conditions [149,183,186]. However, *Synechococcus* PCC 7002 requires vitamin B12 (cobalamin) to grow [146], unlike *Synechocystis* PCC 6803 and *Synechococcus* PCC 7942 [147]. Thus, the cost of vitamin B12 supplementation should be considered when *Synechococcus* PCC 7002 is to be used for biotechnological purposes requiring large-scale cultures. The vitamin B12 auxotrophy of *Synechococcus* PCC 7002 is due to the fact that it uses a cobalamin-dependent methionine synthase (MetH) for the synthesis of methionine, though it cannot synthesize cobalamin de novo. Recently, a cobalamin-independent methionine synthase *metE* gene from *Synechococcus* PCC 73109 was expressed in *Synechococcus* PCC 7002 to relieve its cobalamin auxotrophy [187], but this modified *Synechococcus* PCC 7002 sub-strain has been little employed yet.

All three model cyanobacteria are growing well on ammonium and nitrate, the usual nitrogen sources for cyanobacteria. In addition, both *Synechocystis* PCC 6803 and *Synechococcus* PCC 7002 can grow on urea (a frequent pollutant) as the sole nitrogen source [188,189,190], unlike *Synechococcus* PCC 7942 [190]. Furthermore, *Synechococcus* PCC 7002 and *Synechocystis* PCC 6803 are salt resistant (*Synechococcus* PCC 7002 is a marine strain), unlike the freshwater strain *Synechococcus* PCC 7942 [191]. Thus, *Synechococcus* PCC 7942 is not a suitable cell factory for future projects aiming at the photosynthetic production of chemicals in waters polluted by urea and/or salt to save the costs of potable waters. 

As iron is an essential enzyme cofactor for oxygenic photosynthesis, cyanobacteria utilize multiple strategies to maintain iron levels within a desired range. One of them is the synthesis, export, and re-import of ferric ion chelators called siderophores. Both *Synechococcus* PCC 7942 and *Synechococcus* PCC 7002 can synthesize siderophore, unlike *Synechocystis* PCC 6803 that can only import the siderophore produced by other organisms [192]. These three model cyanobacteria have other metabolic differences. The RbcX chaperone operating in assembly of the CO_2_-fixing RubisCO enzyme is essential in *Synechococcus* PCC 7002 [193], whereas it is dispensable in *Synechococcus* PCC 7942 [194] and *Synechocystis* PCC 6803 [195]. In addition, *Synechocystis* PCC 6803 has four flavodiiron proteins (Flv1−Flv4) [196], which function as heterodimers Flv1/3 and Flv2/4. Flv1/3 catalyzes the NAD(P)H-driven reduction in oxygen to water on the acceptor side of PSI [196,197,198], while Flv2/4 operates both in the photoprotection of the photosystem II [199] and in an oxygen-dependent alternative electron flow [200]. Unlike *Synechocystis* PCC 6803, both *Synechococcus* PCC 7942 and *Synechococcus* PCC 7002 possess only Flv1/3, not Flv2/4 [201]. 

Finally, both *Synechocystis* PCC 6803 and *Synechococcus* PCC 7002 secrete extracellular polymeric substances mainly composed of exopolysaccharides, which act in the formation of biofilm and the protection against salt and metals stresses [202,203,204,205], unlike *Synechococcus* PCC 7942 that do not normally form biofilms [206].

### 6.3. Comparison of the Stress-Responsive Glutathione and DNA Repair Systems of the Model Cyanobacteria Synechocystis PCC 6803, Synechococcus PCC 7942, and Synechococcus PCC 7002

Attesting the importance of the glutathione system for the tolerance to photo-oxidative stress and cell detoxication, most cyanobacteria possess the glutathione system. Its composition varies depending on the species and the environmental challenges they face. Most cyanobacteria have a glutathione reductase (GR) enzyme as *Synechococcus* PCC 7942, unlike both *Synechocystis* PCC 6803 and *Synechococcus* PCC 7002 [25,45]. Furthermore, *Synechocystis* PCC 6803 and *Synechococcus* PCC 7002 possess three glutaredoxins (Grxs) operating in tolerance to oxidative and metal stresses [34,45,133,134,135], whereas *Synechococcus* PCC 7942 has only two Grxs [25,131]. Another common feature shared by *Synechocystis* PCC 6803 and *Synechococcus* PCC 7002 is the fact that they both have an orange carotenoid protein (OCP) operating in photoprotection, whereas *Synechococcus* PCC 7942 has no OCP [207]. *Synechocystis* PCC 6803 and *Synechococcus* PCC 7942 have a MAPEG-type glutathione transferase enzyme operating in the tolerance to temperature stresses [29], which could occur during culture in open ponds, unlike *Synechococcus* PCC 7002. 

Concerning the also important DNA repair system, which has been poorly studied in cyanobacteria, it is worth noting that the three model species have the following common and specific features. *Synechocystis* PCC 6803 possesses the *recD* gene, which is absent in *Synechococcus* PCC 7002 and *Synechococcus* PCC 7942 [58]. Furthermore, *Synechocystis* PCC 6803 has *recB* and *recJ*, which are duplicated in *Synechococcus* PCC 7002 and *Synechococcus* PCC 7942.

*Synechocystis* PCC 6803 and *Synechococcus* PCC 7942 have the *umuC* and *umuD* genes (*umuC* is duplicated in *Synechocystis* PCC 6803), unlike *Synechococcus* PCC 7002 that has no *umuC* and *umuD* genes [58]. Furthermore, *Synechocystis* PCC 6803 and *Synechococcus* PCC 7942 *recQ*, which is duplicated in *Synechococcus* PCC 7002.

*Synechococcus* PCC 7942 has *mutT* and *mutY*, whereas *Synechococcus* PCC 7002 lacks *mutT* and *Synechocystis* PCC 6803 lacks *mutY* [58]. 

*Synechocystis* PCC 6803 and *Synechococcus* PCC 7002, which can both use exogenous carbohydrates to accelerate their growth [6], possess *lexA*, which encodes a transcription regulator involved in carbon assimilation, not DNA repair [90]. In contrast, the obligate photoautotroph *Synechococcus* PCC 7942 has no *lexA* [58].

*Synechocystis* PCC 6803, *Synechococcus* PCC 7002, and *Synechococcus* PCC 7942 have also different features in the simplest DNA repair system, which removes only the base-modifying agent in one single step catalyzed by the AlkB demethylase, the Ogt alkyltranferase, and the Phr photolyase. All three model cyanobacteria possess *phr*, whereas *Synechocystis* PCC6803 has *alkB* but not *ogt*, *Synechococcus* PCC7942 has *ogt* (duplicated) but not *alkB*, and *Synechococcus* PCC7002 has neither *alkB* nor *ogt* [58]. 

### 6.4. Comparative Analysis of the Growth and Response to Stresses of the Three Model Cyanobacteria Synechocystis PCC 6803, Synechococcus PCC 7002, and Synechococcus PCC 7942

To better characterize the common and different physiological features exhibited by these three model cyanobacteria, it would be very interesting to perform simultaneous analyses and comparisons of their growth and tolerance to stresses in the same laboratories. This task is of high interest for not only basic science but also for biotechnological projects, which use of robust cell factories capable to withstand the environmental challenges imposed by the industrial process and the possible toxicity of the intended products. So far, a very limited number of comparative studies have been carried out. *Synechocystis* PCC 6803 was shown to be more tolerant than *Synechococcus* PCC 7942 to UV (and gamma radiations) [90] and to the undecane hydrocarbon [208]. Reciprocally, *Synechococcus* PCC 7942 appeared to be more tolerant to chromate than *Synechocystis* PCC 6803, possibly because the sulfate transporters of *Synechococcus* PCC 7942 have lower affinity to chromate than those of *Synechocystis* PCC 6803 [209]. Furthermore, chromate generated more ROS (reactive oxygen species) in *Synechocystis* PCC 6803, as compared to *Synechococcus* PCC 7942, likely because *Synechocystis* PCC 6803 has intrinsic levels of superoxide dismutase, catalase and 2-Cys-peroxiredoxin than *Synechococcus* PCC 7942 [210]. Additionally, interestingly, *Synechocystis* PCC 6803 and *Synechococcus* PCC 7002 were reported to be more tolerant to ethanol than *Synechococcus* PCC 7942 [208,211]. 

Another important aspect to study is the genetic stability of the recombinant cyanobacteria generated for applied research. It has been reported that many engineered cyanobacteria appeared to be genetically unstable [58]. In one *Synechocystis* PCC 6803 recombinant strain, the instability was caused by the transposition of an IS5 insertion sequence leading to the inactivation of a regulatory gene [56]. Thus, it is of interest to note that the genomes of *Synechococcus* PCC 7942 and *Synechococcus* PCC 7002 are predicted to contain one and approximatively 10 transposase encoding genes, whereas *Synechocystis* PCC 6803 possesses a single transposase gene. 

### 6.5. As Observed in Synechocystis PCC 6803, the Sub-Strains of a Single Cyanobacterium Cultivated in Various Laboratories Can Have Different Behaviors

Another level of complexity is the un-surprising finding that a single cyanobacterium cultivated for some times in different laboratories tend to develop genetic and physiological differences from one laboratory to another. In the case of *Synechocystis* PCC 6803, originally isolated from a fresh water lake in California [146] and deposited in both the American Type Culture Collection (strain number ATCC 27184) and the Pasteur Culture Collection (strain number PCC 6803), it was reported that several sub-strains cultivated in different laboratories varied in genotypes and/or phenotypes. As compared to the original genome sequence [48], several specific mutations were identified in genes related to photosynthesis, transport, or motility [212,213,214,215,216,217,218]. Furthermore, variability in genome copy number was also observed [162]. Moreover, phenotypic variations were also reported including photosynthesis [217], cell size [219], motility [212,218], capability to grow on glucose as the carbon source [212,220], or resistance to temperature and salt (NaCl) stresses [221].

## 7. Genetic Characteristics of the Model Cyanobacteria *Synechocystis* PCC 6803, *Synechococcus* PCC 7942, and *Synechococcus* PCC 7002

Shortly after their selection based on their robustness and simple (unicellular) morphologies [146,147] *Synechocystis* PCC 6803, *Synechococcus* PCC 7942, and *Synechococcus* PCC 7002 were found to be naturally competent for genetic transformation (see below). 

### 7.1. Synechocystis PCC 6803, Synechococcus PCC 7942, and Synechococcus PCC 7002 Are Naturally Competent for Genetic Transformation

Natural competence for transformation of prokaryotes refers to their capability to take up DNA from their environment and incorporate it into their own genome. Originally, antibiotic resistant mutants, generated after UV or chemical mutagenesis, were selected, and their genomic DNA was isolated and used to transform wild type cells selecting for antibiotic resistance. Three naturally transformable species have emerged from such studies, namely, *Synechococcus* PCC 7942 [222], *Synechococcus* PCC 7002 (*Agmenellum quadruplicatum* PR6) [223,224], and *Synechocystis* sp. PCC 6803 [225]. In *Synechococcus* PCC 7942, the efficiency of transformation was found to decrease at pH lower than 7.0 or temperature ≥40 °C, two conditions being also unfavorable for growth. Furthermore, transformation was best effective with cells reaching the transition from the first to the second exponential phases of growth, supporting the notion that transformation depends upon the physiological stage of the culture [183,226]. In addition, transformation appeared to be more efficient when expression of the transferred genes was allowed for 24 h on non-selective solid medium, prior to introducing the selective antibiotic underneath the agar of the plate for its slow diffusion toward the cells for gentle selection of the transformants [183]. Additionally, interestingly, the transformation to *Synechocystis* PCC6803 was reported to be strongly stimulated after the deletion of the exonuclease *recJ* gene [227]. Recently, the natural competence for transformation was shown to involve pilus appendages [228] and to be regulated by the circadian clock [229].

In *Synechococcus* PCC 7002, it was shown that single-stranded DNA cannot transform competent cells. Furthermore, cells in the stationary phase of growth or deprived of nitrogen or light before exposure to donor DNA tend to lose their competence for transformation. In contrast, significant improvement in transformation frequency were achieved by increasing the nitrate content of the culture medium or lowering the temperature from 39 °C (the optimal temperature for growth) to 30 °C before exposure to donor DNA [230].

Later, it was established in *Synechococcus* PCC 7942 [231], *Synechococcus* PCC 7002 [232], and *Synechocystis* PCC 6803 [161,163,233] that the donor DNA is integrated in the chromosome, or an endogenous plasmid (see below) of the recipient cells, through homologous recombination (double crossing-over or gene conversion) occurring on each side of the transferred DNA sequence in the region of homology between both the donor DNA and the recipient DNA. This process, similar to what had been described earlier in *Bacillus subtilis* [234], was extensively used for deleting cyanobacterial genes (or a part of them) through their targeted replacement by an easy selectable antibiotic resistance marker. Soon after its discovery, natural transformation of cyanobacteria was used in pioneering in vivo analyses of proteins involved in photosynthesis and/or stress resistance, which were performed with *Synechocystis* PCC 6803 [235,236,237,238,239,240], *Synechococcus* PCC 7942 [241,242,243,244,245], and *Synechococcus* PCC 7002 [246,247]. 

The molecular mechanism of natural transformation has been recently described in *Synechocystis* PCC 6803 [248]. First, DNA uptake from the environment is mediated by binding to the (type IV) pili appendages, which are also involved in cell adhesion and biofilm formation as well as twitching motility. During pilus retraction, the DNA is pulled into the periplasmic space, where one DNA strand is degraded while the other is translocated further across the cytoplasmic membrane by the Com (competence) proteins. The single-stranded DNA arrived in the cytoplasm is protected from nucleases and incorporated into the genome of recipient cells via homologous recombination.

### 7.2. Interest and Limitation of the Polyploidy of Synechocystis PCC 6803, Synechococcus PCC 7942, and Synechococcus PCC 7002

In cyanobacteria, genomic modification is a time-consuming process because these organisms are polyploid. For example, *Synechococcus* PCC 7942, *Synechococcus* PCC 7002 and *Synechocystis* PCC 6803 harbor about 2–5 and 10–12 chromosome copies per cell [161,175,176,177]. Thus, to create a homoploid mutant, a segregation procedure must be applied to ensure that all chromosome copies in the transformants carry only the modified DNA. This requires multiple rounds of culture streaking in the presence of the selective antibiotic, which can last several weeks. However, the polyploidy of cyanobacteria is not a merely negative trait. It allows to study genes that are essential to cell life. In such cases, we obtain the corresponding heteroploid mutants, which possess both mutant and WT chromosomes copies (with and without the studied vital gene). Such mutants survive because they retain a limited but sufficient amount of the studied essential protein, and they usually have a phenotype different from the wild-type strain. This difference often serves as a guide to infer a role of the studied crucial protein.

### 7.3. Utilization of Neutral Genome Sites for Gene Manipulation in Synechocystis PCC 6803, Synechococcus PCC 7942, and Synechococcus PCC 7002

Transformation has been used extensively for the introduction of endogenous or heterologous genes into neutral chromosomal or plasmid sites, i.e., loci that can be disrupted with no negative effect on cellular viability. Many neutral sites localized inside a dispensable gene or intergenic region have been identified in the chromosome or in an endogenous plasmid of *Synechocystis* PCC 6803 [163,249,250,251,252], *Synechococcus* PCC 7942 [253,254,255] and *Synechococcus* PCC 7002 [256,257,258]. These neutral cloning sites were frequently used for cloning and expression of endogenous, or heterologous, genes involved in cell metabolism, stress responses, or the engineering of recombinant strains for the photosynthetic production of chemicals (see Table 1, Table 2 and Table 3). 

In *Synechocystis* PCC 6803, the frequently used neutral loci are (i) the gene *slr0168* or the intergenic region between *slr2030* and *slr2031*, which have no known function; (ii) the *cpcB* gene involved in the synthesis of the photosynthetic pigment phycocyanin; and (iii) the three *psbA* genes (Table 1). Although *psbA1* is not expressed [235,259], *psbA2* and *psbA3*, encoding the D1 protein subunit of the photosystem II, are expressed and dispensable, but they cannot be inactivated simultaneously [259]. The deletion of *psbA2* gene is compensated by an up-regulation of *psbA3* [260]. Neutral sites on the endogenous plasmids pCC5.2 and pCA2.4 have also been identified and evaluated for genetic integration and expression [251,261]. One neutral site on pCC5.2 was used for cloning the limonene synthase genes from *Mentha spicata* or *Citrus limon*, which directed the production of limonene [262]. Interestingly, the production of fluorescent proteins directed from neutral sites in pCC5.2 or pCA2.4 were, respectively, 14- or 100-fold higher than those observed after chromosomal integration [249,261,263,264]. These data are consistent with the finding that the endogenous small plasmids have a higher copy number than the chromosome [168]. These findings suggest that to be well-expressed genes should be cloned preferentially in a small endogenous plasmid than in the chromosome, but this assumption remain to be verified with other genes. 

**Table 1:** Literature on the utilization of neutral chromosomal cloning sites in *Synechocystis* PCC 6803.

Neutral Site and Objective of the Gene Manipulation	References
***psbA1*: a silent gene**	[235]
Photoproduction of extra bicarbonate transporters to increase biomass	[265]
Increase carbon import to improve growth	[266]
Photoproduction of isobutanol	[267]
***psbA2* (slr1311): gene encoding the D1 protein of the PSII**	[259]
Photoproduction of zeaxanthin	[268]
Analysis of a thioredoxin-interacting LuxR-like regulator	[269]
Photoproduction of beta-caryophyllene	[270]
Photoproduction of D1 protein of the PSII	[271]
Photoproduction of polyhydroxybutyrate (PHB) biodegradable bioplastics	[272]
Photoproduction of isoprene	[273,274,275,276,277]
Photoproduction of lipids	[278]
Photoproduction of aromatic amino-acids	[279]
Photoproduction of tryptophan	[280]
Analysis of endogenous flavodiiron proteins	[281,282]
***cpcB*: phycocyanin synthesis gene**	
Photoproduction of isoprene	[283,284]
Photoproduction of geranyllinalool	[285]
***glpK*, the gene encoding the glycerol kinase enzyme**	
Photoproduction of ipid	[286]
**Combination *psbA2* and *cpcB***	
Cloning of various genes for the photoproduction of isoprene	[287]
Photoproduction of β-phellandrene	[288,289,290,291,292,293]
**slr0646 encoding the PBP5 dispensable penicillin binding protein**	[294]
Photoproduction of the p-coumaric acid	[295]
***ndhB* (sll0223): encoding subunit 2 of the NAD(P)H-dehydrogenase**	
Analysis of the circadian expression of the DnaK (heat-shock protein) encoding gene	[296]
Construction of bioluminescent reporter strains for metal detection	[297]
Analysis of the light regulation of the photosystem I genes	[298,299]
***slr0168***	[220]
Analysis of tolerance to stresses	[300,301,302]
Analysis of endogenous and heterologous Fe- or Cu/Zn superoxide dismutase	[302]
Promoter analysis	[303]
Analysis of fatty-acids synthesis	[304]
Photoproduction of ethylene	[305,306]
Photoproduction of lactate	[307,308,309,310]
Photoproduction of 2,3-butanediol	[311]
Photoproduction of sucrose	[312]
Development of a marker-less gene replacement tool	[313]
Photoproduction of fatty alcohol	[314]
Photoproduction of isoprene	[275]
Photoproduction of ethanol	[315,316,317]
Photoproduction of glycerol	[318]
Photoproduction of *n*-butanol	[319]
Analysis of the regulation of Rubisco	[320]
Promoter analysis	[321]
Photoproduction of 1,2-propanediol	[322]
Analysis of alka(e)ne turnover	[323]
Photoproduction of mannitol	[324]
Photoproduction of bisabolene	[325]
***slr0168* and *slr1193***	
Photoproduction of ethanol	[326]
***slr0168* and *slr1556***	
Photoproduction of alkanes	[327]
***ndhB* and *slr0168***	
Photoproduction of ethanol	[315]
***psbA1* and *slr0168***	
Analysis of promoters	[328]
***psbA2* and *slr0168***	
Photoproduction of fatty-acids	[329]
Analysis of the cyanobacterial iron superoxide dismutase SOD	[302]
Photoproduction of ethylene	[330]
***phaCE* genes operating in the synthesis of polyhydroxybutyrate (PHB) biodegradable bioplastics**	[331]
Photoproduction of acetone	[332]
**Intergenic region between *slr2030 andslr2031***	
Analysis of glutathione synthesis	[333]
Analysis of heme oxygenase encoding genes	[334]
Photoproduction of poly-hydroxybutyrate (PHB) biodegradable bioplastics	[335]
Analysis of Flv3 flavodiiron protein	[336]
Photoproduction of pinene	[337]
Analysis of promoters and ribosome binding sites	[338]
Development of the CRISPR technologies for gene deletion or silencing	[339]
Photoproduction of ethylene	[340]
***slr0846* and *slr2030*-*slr2031* intergenic region**	
Photoproduction of glutamate, linalool, and valencene	[341]
**Intergenic regions between *sll0821-slr0846* and *slr2030-slr2031***	
Photoproduction of limonene	[342]
***slr0168* and *slr1704-sll1575* intergenic region**	
Photoproduction of 3-hydroxypropionic acid	[343]
***slr0168*, *psbA2, and slr2030-Slr2031* intergenic region**	
Photoproduction of the manoyl oxide terpene	[344]
Photoproduction of ethanol and butanol	[345]
**Intergenic regions between** ***slr1495*-*sll1397*, *slr1362*-*sll1274*, *slr1828*-*sll1736*, and *slr1992*-*phaA2***	
Photoproduction of 3-hydroxybutyrate the precursor of the synthesis of PHB	[346]

The chromosomal cloning sites, written in bold cases are highlighted in grey color. The *cpcB* gene operates in the synthesis of the phycocyanin pigment. The *glpK* gene encodes the glycerol kinase. *slr0646* encodes the PBP5 dispensable penicillin binding protein [294]. The gene *ndhB* (sll0223) encodes a subunit of a NAD(P)H-dehydrogenase enzyme. The gene *slr0168* has no known function [220]. The *phaCE* genes operate in the synthesis of polyhydroxybutyrate (PHB) biodegradable bioplastics [331]. While *psbA1* is a silent gene [235], its homologue *psbA2* (slr1311) encodes the D1 protein of the photosystem II [259].

In *Synechococcus* PCC 7942 (Table 2), the most frequently employed chromosomal neutral sites are NSI (GenBank accession n° U30252), NSII (GenBank accession U44761) [253]), and NSIII (GenBank accession ABB56771.1) [254,255].

**Table 2:** Literature on the utilization of neutral chromosomal cloning sites in *Synechococcus* PCC 7942.

Neutral Site and Objective of the Gene Manipulation	References
**NSI**	
Photoproduction of 1,2-propanediol	[347]
Photoproduction of glycerol	[348]
Photoproduction of lactate	[349]
Photoproduction of succinate	[350]
Photoproduction of ethylene	[351]
Photoproduction of ethanol	[352]
**NSII**	
Photoproduction of free fatty acids	[353,354,355]
Photoproduction of B12 vitamin	[356]
**NSIII**	
Photoproduction of isobutyraldehyde	[357]
Photoproduction of carboxysome proteins	[358]
**NSI and NSII**	
Analysis of the circadian rhythm	[359]
Photoproduction of isobutyraldehyde	[360]
Photoproduction of 1-butanol	[361,362]
Analysis of carboxysomes	[363,364]
Photoproduction of isopropanol	[364]
Photoproduction of isobutanol	[365]
Photoproduction of 3-hydroxypropionic acid	[366]
Photoproduction of 1,3-propanediol	[367]
Photoproduction of amorphadiene and squalene	[368]
Photoproduction of limonene	[369]
Photoproduction of acetone	[370]
Photoproduction of isoprene	[371]
Analysis of gene-expression control systems	[372]
Photoproduction of 2,3-butanediol	[373]
Photoproduction of farnesene	[374,375]
Photoproduction of lactate	[376]
**NSI and NSIII**	
Photoproduction of biomass and sucrose export	[377]
Photoproduction of a synthetic CO_2_-fixing photorespiratory bypass	[378]
Photoproduction of 2,3 butanediol	[379]
Photoproduction of amorphadiene or squalene	[380]
**NSI, NSII, and NSIII**	
Overproduction of transporters to facilitate sugar export	[254]
Promoter analysis	[381]
Photoproduction of polyketides	[382]
Analysis of the influence of pilus biogenesis on the natural transformation	[229]
***psbA1***	
Photoproduction of ethylene	[383]
***glgc***	
Photoproduction of isobutanol	[366]
**Intergenic region *Synpcc7942_0893* and *Synpcc7942_0894***	
Photoproduction of 2,3-butanediol	[384]

The chromosomal cloning sites, written in bold cases are highlighted in grey color.

In *Synechococcus* PCC 7002, the few neutral cloning sites employed (Table 3) are mainly the chromosomal genes *glpK*, which had been thought for some time to harbor a frameshift mutation preventing the production of a functional glycerol kinase enzyme [385], and *acsA*, which encodes an acetyl-CoA ligase, the inactivation of which conferred resistance to (3-hydroxy)propionate [386].

**Table 3:** Literature on the utilization of chromosomal neutral cloning sites in *Synechococcus* PCC 7002.

Neutral Cloning Sites	Objective of the Gene Manipulation and References
*glpK* (SYNPCC7002_A2842) encoding the glycerol kinase [385,386]	Removal of carboxysomes for containment of genetically modified strains [387]
*acsA* gene (SYNPCC7002_A1838) encoding an acetyl-CoA ligase and *glpK*	Analysis of an organic acid-based counter selection system [386]
*acsA* and *glpK*	Analysis of promoters and ribosome binding sites [388]
Integration between SYNPCC7002_A0935 and SYNPCC7002_A0936	Photoproduction of bisabolene and limonene [388]
*glpK* and *desB* (SYNPCC7002_ A0159 encoding a ω3 acyl-lipid desaturase [389] and integration between SYNPCC7002_A0935 and SYNPCC7002_A0936	Development of genetic tools [390]
*glpK* and integration between A0935-A0936	Engineering a strain for melamine degradation [391]
Intergenic regions between SYNPCC7002_A0932 and SYNPCC7002_A0933, SYNPCC7002_A1202 and SYNPCC7002_A1203, SYNPCC7002_A1778 and SYNPCC7002_A1779	Development of genetic tools [257]
SYNPCC7002_A1838, SYNPCC7002_A2542, and SYNPC- C7002_A2842	Photoproduction of L-lysine [392]

The inactivation of the glycerol kinase *glpK* gene (SYNPCC7002_A2842), which was mistakenly annotated as having a frameshift mutation preventing the production of a functional protein [385], has no influence on the physiology of *Synechococcus* PCC 7002 [386]. Similarly, the inactivation of *desB* (SYNPCC7002_ A0159, ω3 acyl-lipid desaturase) has no detrimental influence at temperature above 22 °C [389]. The inactivation of the *acsA* gene (SYNPCC7002_A1838) encoding an acetyl-CoA ligase confers resistance to 3-hydroxypropionate and propionate [386].

### 7.4. Development of Transformable Shuttle Vectors Based on the Endogenous Plasmids of Synechocystis PCC 6803, Synechococcus PCC 7942, and Synechococcus PCC 7002

Because transformation and autonomously replicating plasmids have played a crucial role for gene manipulation in *Escherichia coli*, several groups tried to introduce an *E. coli* plasmid (pBR322 and its pUC derivatives) into cyanobacteria by transformation. All attempts were unsuccessful [163,178,231], in spite of a single report [393] that was never confirmed thereafter. These findings indicated that these *E. coli* plasmids are not able to replicate in cyanobacteria. Consequently, chimeric plasmids capable to replicate both in *E. coli* and a transformable cyanobacteria were constructed by cloning a small (cryptic) cyanobacterial plasmid (or a part of it) into an *E. coli* plasmid [394]. This approach was initiated in *Synechococcus* PCC 7942 using its smaller 7.84 kbp endogenous plasmid [182,395]. Then, the transformation efficiency was increased by plating the transformation mixture (recipient cells plus transforming DNA) on solid medium and incubating the plate in standard condition for 24 h prior to adding the selective antibiotic underneath the agar of the plate [183]. The transformation was more efficient when the recipient cyanobacterial cells were taken at the transition from the first to the second exponential growth phases [183], similarly to what has been observed for the transformation with linear chromosomal DNA [226]. The influence of the growth phase on transformation, which was not confirmed by other workers [396], was also observed in *Synechocystis* PCC 6803 [163]. 

*Synechococcus* PCC 7942 was found to be transformed more efficiently by chimeric-plasmid DNA isolated directly from this cyanobacterium rather than from *E. coli* [182]. This was explained by assuming that *Synechococcus* PCC 7942 and *E. coli* DNA are differently modified, for instance, by *dam*- or *dcm*-like DNA methylation systems [182]. Similarly, *Synechococcus* PCC 7002 was more efficiently transformed by its chimeric plasmid when it had been isolated directly from this host rather than from *E. coli* [397]. Furthermore, the elimination from this *Synechococcus* PCC 7002 biphasic plasmid of the *AvaI* restriction site (cleaved by the *AvaI*-isoschizomer *AquI* endonuclease of *Synechococcus* PCC 7002) strongly increased the efficiency of transformation to *Synechococcus* PCC 7002 [397]. The importance of DNA modification for genetic transformation and cell fitness of cyanobacteria were firmly established in *Synechocystis* PCC 6803 [398,399,400,401].

The biphasic plasmids autonomously replicating in *E. coli* and a specific cyanobacterium were improved by several groups through the addition of restriction sites for facile gene cloning and/or several antibiotic-resistance genes for effective selection in *Synechococcus* PCC 7942 [402,403,404], *Synechocystis* PCC 6803 [163], and *Synechococcus* PCC 7002 [405]. These shuttle vectors were used for complementation analyses selecting for both the antibiotic resistance of the vector and the wild-type phenotype [406]. This approach allowed the identification and analysis of genes encoding the key stress-defense proteins RecA [405] and the Mn SOD [407]. One *Synechocystis* PCC 6803 shuttle vector served for analyzing the activity of several *E. coli* promoters, such as the *tac* promoter which appeared to be as effective in *Synechocystis* PCC 6803 as in *E. coli* [408]. This promoter-probe vector was also used to show that the lambda phage *Ci*_857_ gene (encoding a temperature sensitive repressor) and associated P*_R_* promoter can be employed for strong and tight temperature-controlled gene expression [408]. Latter these gene expression devices were also shown to work well in other cyanobacteria [409,410]. 

The *Synechococcus* PCC 7002 and *Synechococcus* PCC 7942 shuttle vectors were employed for the production of heterologous larvicidal proteins in cyanobacterial cells, which turned out to be toxic when ingested by mosquito larvae [411,412]. Other studies reported the cloning of heterologous genes in *Synechococcus* PCC 7942 to increase its resistance to cadmium [413] or salt [414,415]. Similarly, the *Zymomonas mobilis* genes encoding the pyruvate decarboxylase (*pdc*) and alcohol dehydrogenase II (*adh*) were cloned into a *Synechococcus* PCC 7942 shuttle vector to engineer an ethanol producer [416]. The same strategy of cloning heterologous genes into a shuttle vector was used to generate a *Synechococcus* PCC 7942 recombinant strain for the production of ethylene [417,418,419]. Chimeric shuttle vectors were also used to overexpress endogenous metabolic genes to improve CO_2_-fixation and/or biomass production in *Synechococcus* PCC 7942 [420]. 

### 7.5. The Chimeric Shuttle Vectors Based on an Endogenous Cyanobacterial Plasmid Tend to Have a Narrow-Host-Range of Replication

The abovementioned biphasic shuttle plasmids based on a cyanobacterial replicon appeared to replicate in one or a few genetically manipulable cyanobacteria. For examples, the *Synechococcus* PCC 7942 chimeric plasmids could not transform *Synechocystis* PCC 6803 [163], and reciprocally, the *Synechocystis* PCC 6803 shuttle vectors could not transform *Synechococcus* PCC 7942 [163]. In contrast, a shuttle vector based on an endogenous plasmid from *Synechococcus* PCC 7942 was shown to replicate in *Anabaena* PCC 7120, but not in *Synechocystis* PCC 6803 [421].

Plasmid vectors that can be transferred to, but cannot replicate in, a host are interesting for transporting DNA that must either transpose (e.g., a transposon, useful for random mutagenesis) or by homologous recombination in order to be stably maintained. 

### 7.6. The Development of Autonomously Replicating Vectors Derived from the Broad-Host-Range Conjugative Plasmid RSF1010 Has Boosted the Genetics of Cyanobacteria

The interest of chimeric plasmid vectors that uses two narrow-host-range replicons originating from *E. coli* and a cyanobacterium is limited by the fact that they can shuttle only from *E. coli* to mostly the corresponding cyanobacterium. Such narrow-host-range vectors are not suitable for rapid tests and comparisons of a gene function in diverse cyanobacterial hosts to select (and engineer) a cyanobacterial chassis for an effective photosynthetic production of an industrially interesting chemical. Thus, several groups turned their attention to the naturally-occurring RSF1010 plasmid [422], a member of the incQ incompatibility group that replicates in a wide range of Gram-negative bacterial genera (cyanobacteria are also Gram-negative), including *Acetobacter*, *Acinetobacter*, *Agrobacterium*, *Alcaligene*, *Azotobacter*, *Escherichia*, *Klebsiella*, *Methylophilus*, *Providencia*, *Pseudomonadales*, *Rhizobiaceae*, *Rhodopseudomonas*, and *Salmonella* SP- and *Serratia*. The value of RSF1010 as a shuttle vector is further enhanced by its ability to be transferred by conjugation from an *E. coli* strain also carrying a self-transmissible (incP group) plasmid, such as RP4. The RSF1010-derived plasmids can be used to carry novel genetic information to those bacteria that are not capable of transformation [423,424,425]. 

RSF1010 and its derivatives were shown to be efficiently transferred by conjugation from *E. coli* to cyanobacteria where they replicate autonomously, even though they contain no cyanobacterial replicon. These cyanobacteria were, namely, *Synechocystis* PCC 6803 [426], *Synechococcus* NKBG15041C, *Pseudanabaena* NKBG040605C [427], *Synechocystis* PCC 6714, *Synechococcus* strains PCC7942 and PCC6301 [428], *Thermosynechococcus elongatus* PB1 [429], marine *Synechococcus* strains sp. WH7803, WH8102 and WH8103 [430], *Gloeobacter violaceus* PCC 7421 [431,432], *Prochlorococcus* MIT9313 [433], *Nostoc* (*Anabaena*) PCC 7120, *Nostoc punctiforme* ATCC29133 (also registered as PCC 73102) [255,434,435], *Leptolyngbya* BL0902 [436] and *Cyanothece* PCC 7425 [410]. 

Shortly after, the *Synechocystis* PCC6803 pioneering report [426], RSF1010 was used for the development of the first conjugative plasmid vector for promoter analysis [409] and regulated protein production in cyanobacteria [437]. The promoter-probe vector used the promoter-less chloramphenicol-acetyl-transferase (*cat*) gene as the reporter. When expressed by a studied promoter, *cat* directs the production of the CAT enzyme, the activity of which can be monitored by a spectrophotometric assay and confers the resistance to chloramphenicol [409]. This vector was used for the analysis of constitutive or regulated promoters [126,438,439] and references therein. The conditional expression vector [437] harbors the lambda-phage gene *cI_857_* encoding a temperature-sensitive repressor that tightly controls the activity of the otherwise strong *p_R_* promoter. Together these elements allow a tight temperature-controlled expression of the studied genes (no production of the corresponding proteins at temperatures below 30 °C, moderate level at 34–36 °C, and high production at 39–40 °C). This vector and its derivative harboring the *gfp* gene encoding the green-fluorescent reporter protein have been employed for proteins involved in photosynthesis [125], response to stress [34], cell division [440,441,442], and biogenesis of the carboxysome [410]. 

Other RSF1010-derived plasmids vectors have been used to analyze ribosome binding sites and transcription terminators [264], light-emitting proteins GFP, YFP (yellow-fluorescent protein), and luciferase [255,264,432,434]. RSF1010-derived vectors were also employed to analyze the role of carbon stores glycogen and PHB (polyhydroxybutyrate) in the tolerance to stress [443] and systems for the control of gene expression (in *Synechococcus* PCC 7942) [372], as well as various proteins such as the *Synechocystis* PCC 6803 photolyase enzyme PhrA [444] and to improve carbon fixation [445].

RSF1010 derivative plasmids were also employed in many works aiming at producing biotechnologically interesting products including bisabolol and patchoulol [446], ethanol [447], ethylene [306,340,448,449,450], erythritol [451], hydrogen [452,453], isobutanol [454,455], lactate [308], limonene [410], n-butanol [319], and triterpenes (lupeol, marnerol, and hydroxymarnerol) [456].

Interestingly, in *Synechocystis* PCC 6803, it has been shown that RSF1010 and the pCC5.2 endogenous plasmid could be used for cloning, respectively, two pentose phosphate pathway native genes and the limonene synthase genes (lims) from either *Mentha spicata* or *Citrus limon*, which directed the production of limonene [262].

## 8. Interest and Limitation of the CRISPR/Cas Genome Editing Technology

Recently, the CRISPR/Cas system (CRISPR stands for clustered regularly interspaced short palindromic repeats and Cas for CRISPR-associated endonuclease) has facilitated the way genomes are edited in cyanobacteria, such as *Synechocystis* PCC 6803, *Synechococcus* PCC 7942, *Synechococcus* PCC 7002, *Synechococcus* UTEX 2973, and the filamentous strain *Nostoc* (*Anabaena*) PCC 7120 (for reviews see [80,83,84,339,457]. Briefly, CRISPR/Cas genome editing systems exploit the Class II family of Cas endonucleases, which have a site-specific RNA-guided DNA cleavage activity. As compared to the well-established gene deletions techniques based on homologous DNA recombination, the interest of the CRISPR/Cas system are (i) CRISPR/Cas systems can allow the engineering of non-transformable cyanobacteria, providing they can be manipulated by conjugation; (ii) a marker-less mutation is generated at the DNA target site; and (iii) multiple DNA loci can be modified simultaneously, by co-expressing the appropriate guide RNAs and editing templates. 

The limitation of the CRISPR/Cas technology are the potential toxicity of the Cas DNase and the time required to eliminate the CRISPR/Cas plasmid vector from the generated mutant. However, this curing step can be accelerated by the presence of a negative selection marker in the CRISPR/Cas vector [80]. 

A variant of the CRISPR/Cas system, the CRISPRi (CRISPR interference) system that make use of DNase-inactive variants of Cas, is especially relevant to repress (fully or not) the transcription of studied genes, including the essential ones that cannot be deleted. This strategy was used for the targeted repression of vital genes to arrest growth and increase carbon partitioning and biofuel titers in *Synechocystis* PCC 6803 [345]. The CRISPRi technology was also employed to generate mutants with increased yields of growth and lactate secretion [458].

## 9. Responses to Stresses: The Recent Progress in Omics Technics Are Limited by the Large Number of Genes of Still Unknown Function

Because of their oxygenic photosynthesis, which triggered oxygen-promoted changes in metal availability, and the fact that they colonized most aquatic biotopes of our planet, cyanobacteria have always been challenged by changes in light, metals, and nutrients availabilities [28]. The responses to these stresses have been well studied with omics techniques that measure the changes in abundance of transcripts (transcriptomics), proteins (proteomics), or metabolites (metabolomics). The available genome sequences facilitate the use of transcriptomic and proteomic approaches. 

Initially, transcriptomics focused on *Synechocystis* PCC 6803, because its genome has been the first to be fully sequenced [48] and used to develop the first commercially available DNA microarrays (IntelliGene™ CyanoCHIP; Takara Bio Inc., Shiga, Japan), which only comprised probes for protein-coding genes deposited on a glass support. These CyanoCHIP were used to the transcriptional responses to high light [459], inhibitors of photosynthesis [460], depletion of the LexA regulator [90], salt [461,462,463,464], cold stress [465], acid stress [466,467], heat shock [468,469,470], osmotic stress [465,471,472], oxidative stresses triggered by methyl viologen [473], or H_2_O_2_ [474,475], Cd-, Fe-, or Zn-stresses [474]. 

Later, several genome-wide *Synechocystis* PCC 6803 microarrays were also developed, based on long oligonucleotide probes (60- to 70-mer) spotted on a glass support. Such oligonucleotides-based microarrays circumvented the labor-intensive and error-prone steps of probe amplification and purification. They were used to study cell responses to sulfur starvation [476] and the deletion of the AbrB2 transcription regulator [477].

As an alternative to microarray analyses, which are based on hybridization of mRNA to DNA probes, the direct sequencing of RNA (an approach designated as RNA-Seq) was adapted to cyanobacteria [478]. RNA-Seq rapidly became the standard method for cyanobacterial transcriptomics [250,479]. It revealed that the *Synechocystis* PCC 6803 transcriptome includes more than 4000 transcriptional units, half of which representing small RNAs (sRNAs), which often harbor a small protein-coding sequence of less than 100 amino acid residues, and non-coding RNAs (ncRNAs) [480]. These ncRNAs could not be detected by DNA microarrays that only comprised probes for protein-coding genes. The vast majority of ncRNAs are still uncharacterized, and most of them are antisense transcripts (asRNAs). The phylogenetic conservation of ncRNAs across genomes of relatively distant cyanobacteria and their regulated transcription in response to major stresses, such as, light, iron, carbon, or nitrogen availability, nitrogen starvation [481], and butanol or ethanol stress [482], suggest that many ncRNAs may be involved in regulation [250,479].

Proteomics was also used to study the cyanobacterial responses to stresses, again starting with *Synechocystis* PCC 6803 [483], the genome of which is predicted to contain 3672 putative open reading frames (ORFs, i.e., protein coding sequences). Of these, 3264 and 408 ORFs are located on the chromosome and the seven endogenous plasmids, respectively [484]. Traditionally, two-dimensional polyacrylamide gels (2D-PAGE) and utilization of different fluorescence dyes (difference gel electrophoresis; 2D-DIGE) were employed to estimate concentrations for each protein between stress versus unstressed conditions. Later strategies took advantage of the sensitivity of liquid chromatography (LC), coupled with tandem mass spectrometry (MS), known as LC-MS/MS, for quantitative proteomic analysis, using different tags such as the isobaric tags for relative and absolute quantitation (iTRAQ)-based quantitative proteomics [485,486]. This quantitative technique became the dominant proteomics method for the identification of differentially expressed proteins of *Synechocystis* PCC 6803 [485,486,487,488]. 

These proteome techniques were employed to study the responses to cold [489]; copper [490]; CO_2_ limitation [491]; high light [492]; high or low temperature [469,492,493,494]; high or low pH [495,496,497]; nitrogen-, phosphate, or sulfate-starvations [481,498,499]; metal stress [498,500,501]; salt stress [502,503,504,505,506,507]; and UV-B stress [508]. They also served to study the tolerance of *Synechocystis* PCC 6803 to butanol, ethanol, or hexane biofuels [488,509,510,511,512], as well as to analyze cyanobacterial strains engineered for the production of butanol [511], ethanol [510], hexane [509], or 3-hydroxypropionic acid [513]. 

In addition to the quantification of proteins, proteome methods can be used to identify protein modifications, such as glutathionylation [40], lysine malonylation [514], lysine methylation [515], phosphorylation [516,517,518], which are potentially involved in controlling protein activities.

However, our current understanding of the transcriptome and proteome responses to various challenges is limited by the fact that a large number of the responsive genes or proteins have still an unknown function.

In addition to transcriptomics and proteomics, metabolomics that focuses on low-molecular-weight metabolites provides the most straightforward characterization of metabolic responses to environmental changes. Compared to other omics studies, a few metabolomic research studies have been performed in cyanobacteria, and again *Synechocystis* PCC 6803 has been the most studied model. As many metabolites turn over quickly, fast sampling through fast culture filtration appeared to be very important in metabolomic analyses [519,520]. The combination of gas chromatography or liquid chromatography with mass spectrometry permits quantitative analysis of more than 100 metabolites in cyanobacterial cells. In addition to metabolomics, which seeks comprehensive profiling of predominantly intra-organism compounds, volatilomics assesses those compounds released by an organism: the key components of chemically mediated inter-organismal communication [521,522]. The field of volatilomics grew out as advances in collection methods of volatile organic compounds and gas chromatography coupled with mass spectrometry. 

Finally, in several studies, transcriptomics, proteomics, and/or metabolomics were integrated to better analyze the responses of *Synechocystis* PCC 6803 to environmental conditions [492,497,523,524,525,526,527,528,529], as well as the production of [530] or the tolerance to chemicals [531]. In some cases, it appeared that omics data at different levels do not necessarily correlate a finding that can be explained by regulations occurring at the levels of gene expression [6,93,479,532,533,534] and/or enzyme stability and activity [40,483,527]. 

## 10. Conclusions

Cyanobacteria are a widely-diverse photosynthetic prokaryotes of wide interest for basic and applied sciences. So far, cyanobacterial research has focused primarily on a few models, such as the three unicellular non-nitrogen fixing species *Synechocystis* PCC 6803, *Synechococcus* PCC 7942, and *Synechococcus* PCC 7002, which are straightforward to culture under laboratory conditions, easily amenable to genetic modification and can be frozen for long-term storage. Extensive “omics” data sets and many genetic tools and genome-scale metabolic models (GSM) have been generated to guide the engineering of these model cyanobacteria for the photosynthetic production of biotechnologically interesting chemicals. Interestingly, it has been put forward that GSM should take into account and describe photon absorption and light-shading thereby addressing the challenge of accurately modeling light as a metabolite [535,536]. In addition, GSM should be validated with experimental data obtained after measurement of metabolic fluxes and metabolic pool sizes [80,86,87,536]. However, omics data interpretation and GSM metabolic designs are based on our currently limited understanding of the genotype–phenotype relationships of cyanobacteria. Thus, to generate robust and predictive GSM models of the cyanobacterial metabolism, it is important to continue the analysis of *Synechocystis* PCC 6803, *Synechococcus* PCC 7942 and *Synechococcus* PCC 7002, and increase the efforts to

(i) verify the function of numerous genes that have been annotated merely by sequence analogy with those genes characterized only in intensively studied non-photosynthetic models (*E. coli*, yeast, etc.), which may have a different function in cyanobacteria;
(ii) and analyze the specificity/redundancy of multiple gene families;
(iii) characterize the function of the large number of as yet unknown genes and non-coding RNAs;
(iv) identify the comprehensive set of genes that are essential to the growth of cells incubated in well-defined conditions.

Furthermore, most of the attempts to reprogram *Synechocystis* PCC 6803, *Synechococcus* PCC 7942, or *Synechococcus* PCC 7002 for the photoproduction of chemicals have focused on increasing product synthesis by small-scale cultures growing under laboratory conditions because most academic researchers lack access to large-scale production systems that are necessary to evaluate the potential of engineered strains under realistic industrial conditions. 

Moreover *Synechocystis* PCC 6803, *Synechococcus* PCC 7942, and *Synechococcus* PCC 7002 represent only a limited part of the wide biodiversity of cyanobacteria. This arguably limits fundamental discovery and applied research towards wider commercialization. Thus, new phylogenetically-distant candidate cyanobacteria should be isolated and developed from diverse environments with a robust growth and high tolerance to local conditions, so as to be used as chassis for the photosynthetic production of high-value chemicals in diverse industrial sites. We think that the genetic modifiability of such candidate strains using the conjugative transfer of RSF1010-derived broad-host-range plasmids will be key for such works.

To summarize, we recommend to strengthen the communication between academic researchers, who know well cyanobacteria and can manipulate them, but have a limited access to large photobioreactors and industrial partners, who attempt to use cyanobacteria to produce interesting chemicals at reasonable costs, but often lack knowledge on cyanobacterial genetics, physiology, and metabolism. Moreover, to minimize operation costs we need to develop robust cyanobacteria capable to grow on industrial waters and fumes, in huge photobioreactors, as well as well as efficient technologies to harvest the end products.
